# Validation of the Identification and Intervention for Dementia in Elderly Africans (IDEA) cognitive screen in Nigeria and Tanzania

**DOI:** 10.1186/s12877-015-0040-1

**Published:** 2015-04-25

**Authors:** Stella-Maria Paddick, William K Gray, Luqman Ogunjimi, Bingileki lwezuala, Olaide Olakehinde, Aloyce Kisoli, John Kissima, Godfrey Mbowe, Sarah Mkenda, Catherine L Dotchin, Richard W Walker, Declare Mushi, Cecilia Collingwood, Adesola Ogunniyi

**Affiliations:** Northumbria Healthcare NHS Foundation Trust, North Tyneside General Hospital, North Shields, UK; Institute of Neuroscience, Newcastle University, Newcastle upon Tyne, UK; University College Hospital, University of Ibadan, Ibadan, Nigeria; Mawenzi Regional Hospital, Moshi, Tanzania; Hai District Hospital, Boman’gombe, Kilimanjaro Tanzania; Kilimanjaro Christian Medical University College, Moshi, Tanzania; Institute for Ageing, Newcastle University, Newcastle upon Tyne, UK; Institute of Health and Society, Newcastle University, Newcastle upon Tyne, UK; The Medical School, Newcastle University, Newcastle upon Tyne, UK

**Keywords:** Dementia, Delirium, Screening, Nigeria, Tanzania, Africa, Validation

## Abstract

**Background:**

We have previously described the development of the Identification and Intervention for Dementia in Elderly Africans (IDEA) cognitive screen for use in populations with low levels of formal education. The IDEA cognitive screen was developed and field-tested in an elderly, community-based population in rural Tanzania with a relatively high prevalence of cognitive impairment. The aim of this study was to validate the IDEA cognitive screen as an assessment of major cognitive impairment in hospital settings in Nigeria and Tanzania.

**Methods:**

In Nigeria, 121 consecutive elderly medical clinic outpatients reviewed at the University College Hospital, Ibadan were screened using the IDEA cognitive screen. In Tanzania, 97 consecutive inpatients admitted to Mawenzi Regional Hospital (MRH), Moshi, and 108 consecutive medical clinic outpatients attending the geriatric medicine clinic at MRH were screened. Inter-rater reliability was assessed in Tanzanian outpatients attending St Joseph’s Hospital in Moshi using three raters. A diagnosis of dementia or delirium (DSM-IV criteria) was classified as major cognitive impairment and was provided independently by a physician blinded to the results of the screening assessment.

**Results:**

The area under the receiver operating characteristic (AUROC) curve in Nigerian outpatients, Tanzanian outpatients and Tanzanian inpatients was 0.990, 0.919 and 0.917 respectively. Inter-rater reliability was good (intra-class correlation coefficient 0.742 to 0.791). In regression models, the cognitive screen did not appear to be educationally biased.

**Conclusions:**

The IDEA cognitive screen performed well in these populations and should prove useful in screening for dementia and delirium in other areas of sub-Saharan Africa.

## Background

The prevalence of dementia, alongside other non-communicable diseases (NCDs), is increasing rapidly as populations age globally, with 135.5 million people expected to have dementia by 2050 [1]. The greatest increases are predicted in low- and middle-income countries (LMICs) with 71% of the global total of people with dementia residing in LMICs by 2050 [1]. In sub-Saharan Africa (SSA) the number of cases of dementia is expected to increase from 1.31 million in 2013 to 5.05 million by 2050 [1]. Identification of dementia and other major cognitive impairments in LMICs can be problematic, due to the lack of culturally appropriate validated screening tools. The vast majority of cognitive screening tools in common use worldwide have been developed and validated in high income countries (HIC) and usefulness in LMIC settings is greatly limited by cultural and educational differences. Illiteracy is highly prevalent in older adults in many LMICs, particularly in rural areas. Minimising educational bias in cognitive screening tools would improve their clinical utility. In SSA, perhaps more than in other LMIC settings, this problem is compounded by a serious shortage of specialist clinicians including neurologists, geriatricians and psychiatrists [2,3]. There are 200 times fewer qualified mental health workers per 100,000 population in SSA compared to HICs [4]. One way to overcome this problem is to develop and validate cognitive screening tools suitable for use by non-specialist healthcare workers and clinicians. These tools must be brief, simple to use and have excellent predictive properties. This task-shifting approach is recommended by the World Health Organization (WHO) for other mental and neurological conditions in low-resource settings [5]. We have recently described the development, internal validation and fieldwork testing of the Identification and Interventions for Dementia in Elderly Africans (IDEA) study cognitive screening tool [6]. This screening tool was developed using data collected from 1198 older adults screened for dementia in rural Tanzania, a low-literacy setting. It was subsequently piloted in a follow up cohort with relatively high levels of cognitive impairment. The aim of the current study was to externally validate the IDEA cognitive screen for use in a cohort of older adults in hospital settings in Nigeria and Tanzania.

## Methods

This current study took place as part of the larger IDEA study of dementia in SSA. In Nigeria, the study was approved by the University of Ibadan and Oyo state Ministry of Health research ethics committees. In Tanzania, the study was approved nationally by the National Institute for Medical Research, and locally by Kilimanjaro Christian Medical University College. Written informed consent was obtained from each participant. We obtained a thumbprint for those that could not read or write after the purpose and implications of the study were verbally explained. In cases where patients were unable to give informed consent due to cognitive deficit, written assent was obtained from a close relative.

### Participants and setting

#### Nigerian cohort

##### Outpatient sample

Participants were geriatric patients, aged 65 years and over, seen at the medical outpatient clinic of University College Hospital Ibadan (UCH) during May 2013. UCH is an 850-bed teaching hospital in the city of Ibadan, Oyo state, western Nigeria. The city has a population of approximately 3 million people. Patients were included if they consented to participate and were 65 years or older.

#### Tanzanian cohort

Mawenzi Regional Hospital (MRH) in Moshi is a government hospital with approximately 200 beds and provides care for around 300 outpatients per day. The hospital serves an urban and rural population of around 100,000 people. Those requiring more specialist services are referred to Kilimanjaro Christian Medical Centre, a tertiary referral hospital in Moshi.

#### Outpatient sample

Outpatients were recruited from the geriatric medicine outpatient clinic at MRH. The geriatric clinic offers a free-of-charge service for those able to demonstrate that they are aged 60 years or over, usually with a letter from their village committee. All attendees at the clinic aged 65 years or over were invited to take part. Screening was conducted daily for a four-week period during October and November 2013. Screening did not take place on public holidays and weekends, as the clinic was closed. Due to resource limitations, a stratified sample of outpatients were clinically assessed. A full clinical assessment of all those scoring ≤ 8 on the IDEA screen was completed. A score of ≤ 7 was considered the optimal cut-off for detection of major cognitive impairment, but the higher cut-off was chosen to try to ensure high sensitivity. We aimed to clinically assess a random selection of at least 40% of those who scored > 8 on the screen. Randomisation involved drawing lots.

#### Inpatient sample

All admissions to the medical wards of MRH aged 65 years and over, from 8^th^ October to 20^th^ December 2013 were invited to take part in the study. The ward admission records were consulted daily and a physical check was made of all wards for new admissions. Patients were excluded if they refused to participate, or if the assessing clinician felt they were too unwell to participate.

#### Inter-rater reliability

This was carried out in the outpatient clinic of St Joseph’s Catholic Mission hospital in Moshi, Tanzania. St Joseph’s was chosen for the assessment of inter-rater reliability to ensure that all patients were previously unknown to the raters, thus avoiding the possibility that their scores could be influenced by prior information. All outpatients aged 65 years and over were invited to take part. Screening was carried out by three trained raters (AK, SM or JK), randomly coded A, B and C. To minimise the confounding influence of a training effect, rater A and rater B were each randomly assigned to see half of the patients on the first assessment and the other half on the second assessment. The third assessment was completed by rater C. For the third assessment, not all patients could be followed up due to having been discharged from the clinic. A minimum gap of two days was left between consecutive assessments to minimise carryover effects. Assessments were timed, where possible, to coincide with existing outpatient appointments in order to avoid additional unnecessary travel for participants, some of whom were frail.

### Assessments

At both sites, basic demographic data (age, gender and highest education level) were collected from each participant. In both countries, birth registration is not universal, and many older people do not know their date of birth. Where age was not accurately known, a validated method of estimation based on significant past events was used [7]. The method has been shown to have excellent concordance during validation work in other SSA populations (intra-class correlation coefficient (ICC) 0.87).

#### Cognitive screening

**Figure 1:**
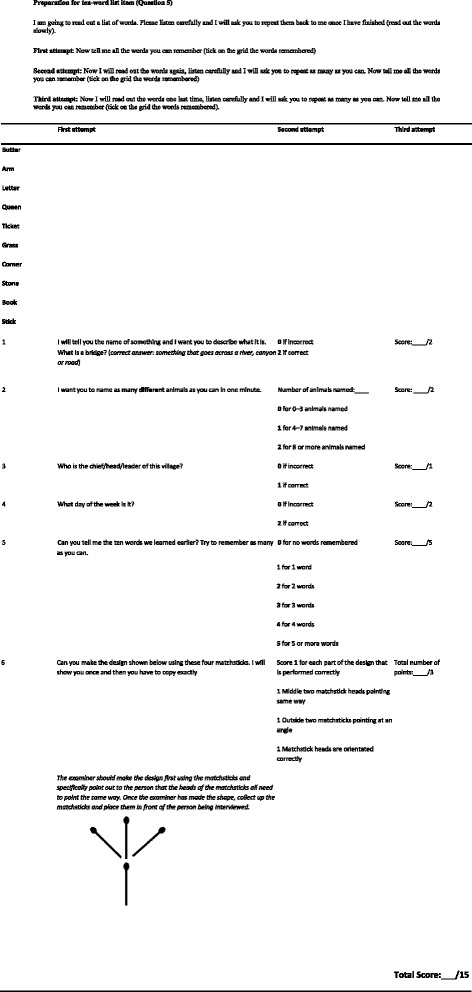
The IDEA cognitive screen in English.

The IDEA cognitive screen has six items derived from existing cognitive assessments used in LMIC [6]. The screen is shown in Figure 1. Items 1–4 are taken from the Community Screening instrument for Dementia (CSI-D) [8]. These involve being able to name a bridge from a description of its use, knowing the day of the week, knowing the name of the village chief/ town mayor/ city governor and naming as many animals as possible in one minute (score 2 for ≥ 8 animals, score 1 for 4–7 animals, score 0 for 0–3 animals). Item 5 is taken from Consortium to Establish a Registry for Alzheimer’s Disease (CERAD) 10-word recall test [9], with recall of 10 common words after 5 minutes delay (score 1 point for each word up to a maximum of 5 points). The sixth item is designed to measure praxis and involves a matchstick design test originally developed by Baiyewu et al. [10], with scores ranging from 0 (no matchsticks placed correctly), to 3 (all four matchsticks placed correctly in the shape of a rake). The maximum possible score is 15 and the minimum 0, with a higher score indicating better cognitive function. The IDEA screen therefore includes delayed recall, orientation, two measures of frontal lobe function, verbal fluency and abstract reasoning, praxis and long-term memory. An assessment of ability for new learning is also possible from performance on the 10-word learning list. No items are included requiring reading, writing, drawing or calculation in order to reduce possible educational bias. The screen was administered in the local language (Yoruba in Nigeria, Swahili or Chagga in Tanzania) with the words from the 10-word list translated into the local equivalent.

In Nigeria, the cognitive screen was administered by a study nurse, prior to formal assessment for cognitive impairment by a doctor. The nurse was therefore blind to the clinical diagnosis at the time of assessment. In Tanzanian inpatients and outpatients, the cognitive screen was completed by one of three assessors: an MSc qualified nurse (AK), occupational therapist (GM) or assistant medical officer (JK). As in Nigeria, to ensure blinding, cognitive screening was conducted prior to clinical assessment by a doctor. After administration, all screening tools were filed and not seen by the doctor completing cognitive and neurological assessment.

#### Clinical assessment

At both sites, participants were assessed clinically for major cognitive impairment. A focused neurological examination, further physical examination (where appropriate) and informant history including usual level of functioning were completed wherever possible. Clinical assessment included bedside cognitive screening designed to cover all major cognitive domains, including orientation, registration and delayed recall, attention and concentration, receptive and expressive language, praxis and frontal lobe function using Luria’s three-step hand position test. A formal mental state examination with completion of the geriatric depression scale (in order to exclude psychiatric disorder as a cause of poor cognitive performance), neurological examination and careful questioning of informants on history and instrumental activities of daily living (IADLs) appropriate to the setting in order to assess functional impairment were also completed. Due to the lack of validated appropriate cognitive screening tools in our setting, greater weight was placed on the informant history and psychiatric and neurological examination than the outcome of bedside cognitive assessment when reaching a clinical diagnosis.

In inpatients, the Confusion Assessment Method (CAM) was completed where there was evidence of cognitive impairment. Care was taken to ensure that the assessing doctor remained blinded to the outcome of screening when completing the clinical assessment.

#### Diagnosis of major cognitive impairment

After interview and assessment, a diagnosis of major cognitive impairment was provided as appropriate by the study doctor at each site (AO and LO in Nigeria and S-MP in Tanzania). Diagnoses of dementia, delirium and other significant mental illness, where present, were based on DSM-IV criteria [11]. Informant histories were extremely useful in attempting to differentiate between dementia and delirium and these were sought wherever possible, by telephone if necessary. In cases of diagnostic difficulty, cases were discussed with a specialist in old age psychiatry and a consensus on the most likely clinical diagnosis reached. Anyone with a diagnosis of dementia or delirium was identified as having major cognitive impairment.

### Sample size

For multivariable analysis, a sample size was chosen that would avoid over-fitting the model. Although estimates vary, a minimum of seven cases per predictor was deemed acceptable. Any model was thought unlikely to contain more than eight predictor variables, and so a minimum sample size for each cohort of 56 was calculated.

### Statistical methods

Statistical analysis was conducted using IBM SPSS statistics version 21 (IBM corporation, Armonk, NY, USA). All data (including age) were not normally distributed and so non-parametric tests (Mann–Whitney U test and chi-squared test) were used. For data analysis, education was dichotomised into some education (attended school) and no education (never attended school). Sensitivity, specificity and likelihood ratio (LR) were calculated. Positive predictive value (PPV) was calculated for Nigerian outpatients and Tanzanian inpatients, but not for Tanzanian outpatients. Since not all screened Tanzanian outpatients were clinically assessed, prevalence, and therefore an accurate PPV, could not be estimated. The area under the receiver operating characteristic (AUROC) curve was used as an overall measure of the performance of the IDEA cognitive screen. Cronbach’s α was calculated to assess the consistency of the screen.

We used regression modeling to investigate whether the IDEA cognitive screen was educationally biased. Major cognitive impairment becomes more common with increasing age and is thought to be more common in women than men and more common in those with no formal education [12,13]. However, these three variables are also confounded with each other, with women tending to be overrepresented in older age groups and, in many areas of SSA, less likely to have attended school than men. To assess the independent influence of age, gender and education on screening performance, univariate and multivariable logistic regression models were developed with screening tool score (dichotomised into ≤ 7 and > 7) as the dependent (outcome) variable. Age, gender, education and the presence of major cognitive impairment were forced into a multivariable model as independent (predictor) variables. Univariate models were initially investigated within each of the three cohorts separately. Given the similarity in the results of the univariate analysis, and to increase statistical power, multivariable models were constructed using the combined data from all three cohorts. Education was dichotomised as no formal education or some formal education and age was split into five-year age bands. Inter-rater reliability was assessed using the ICC and by comparing the level of agreement in terms of clinical decision-making. The significance level was set at 5% and two-tailed tests were used throughout.

## Results

In Tanzania, 97 inpatients were seen, of whom 33 (34.0%) had major cognitive impairment (20 dementia, 13 delirium). Of 108 outpatients seen in Tanzania, 16 (14.8%) scored ≤ 8 and all were clinically assessed. Of the remaining 92 who scored > 8, 43 (46.7%) were randomly selected for clinical assessment, giving a Tanzanian outpatient cohort of 59, of whom 13 (22.0%) had major cognitive impairment. All 13 had dementia, though one person with dementia was also thought to have delirium at the time of assessment and was referred for further investigations. In Nigeria, data were available for 121 outpatients, of whom 12 (9.9%) had major cognitive impairment (all dementia).

Thus, 277 were included in this validation study across all three settings. The median time taken to complete the screen was 10 minutes (inter quartile range: 8 to 12 minutes).

### Demographic data

Age, gender and education level data for those with and without major cognitive impairment are presented in Table 1. In Nigeria, those with major cognitive impairment had significantly higher levels of education than those without major cognitive impairment and in Tanzanian outpatients, those with major cognitive impairment were significantly older than those without major cognitive impairment.

**Table 1:** **Validation of the Identification and Intervention for Dementia in Elderly Africans (IDEA) cognitive screen in 2013: Demographic data**

	**Major cognitive impairment**	**No major cognitive impairment**	**Significance of difference**
**Outpatients Nigeria**			
Number of patients	12	109	
Median age (IQR)	71 (65.3 to 77.5)	70 (67 to 75.5)	U = 619.0, z = −0.305, p = 0.761
Number of females	8 (66.7%)	49 (45.0%)	χ^2^ = 2.045, p = 0.153
Level of education*	None: 0	None: 36 (33.0%)	χ^2^ = 5.619, p = 0.018
	Some: 11 (91.6%)	Some: 67 (61.5%)	
	Not known: 1 (8.3%)	Not known: 6 (5.5%)	
**Outpatients Tanzania**			
Number of patients	13	46	
Median age (IQR)	79.5 (73.3 to 89.8)	72 (67.3 to 78.8)	U = 162.5, z = −2.030, p = 0.042
Number of females	7 (53.8%)	21 (45.7%)	χ^2^ = 2.045, p = 0.153
Level of education	None: 5 (38.5%)	None: 11 (23.9%)	χ^2^ = 0.273, p = 0.601
	Some: 8 (61.5%)	Some: 35 (76.1%)	
**Inpatients Tanzania**			
Number of patients	33	64	
Median age (IQR)	78 (72.5 to 90)	75.5 (70.3 to 81)	U = 846.0, z = −1.601, p = 0.109
Number of females	14 (42.4%)	37 (57.8%)	χ^2^ = 2.068, p = 0.150
Level of education	None: 15 (45.5%)	None: 22 (34.4%)	χ^2^ = 1.407, p = 0.236
	Some: 17 (51.5%)	Some: 42 (65.6%)	
	Not known 1 (3.0%)		

IQR = interquartile range.

U = the test value of the Mann–Whitney U test.

* For data analysis education was dichotomised into some education (attended school for at least a year) and no education (never attended school).

### Performance of the IDEA cognitive screen

Cronbach’s α for the IDEA cognitive screen was 0.807 in Tanzanian inpatients, 0.738 in Tanzanian outpatients and 0.741 in Nigerian outpatients, suggesting it to have an acceptable degree of internal consistency in all three settings.

**Figure 2:**
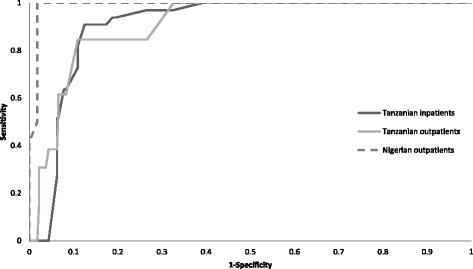
Validation of the Identification and Intervention for Dementia in Elderly Africans (IDEA) cognitive screen in 2013: ROC curves for each cohort.

AUROC curves for each cohort are presented in Figure 2. Across all three settings no one with major cognitive impairment scored more than 10, and only eight scored greater than the suggested cut off of ≤ 7 (three Tanzanian outpatients and one Tanzanian inpatient scored 8, one Tanzanian outpatient scored 9 and one Tanzanian outpatient and two Tanzanian inpatients scored 10). Of 15 people without major cognitive impairment who scored ≤ 7, three had mild cognitive impairment (MCI) and five were aphasic or unable to perform well due to physical or mental illness. Sensitivity, specificity, PPV, LR and AUROC curve data, are shown in Table 2. The AUROC curve was above 0.9 in all settings.

**Table 2:** **Validation of the Identification and Intervention for Dementia in Elderly Africans (IDEA) cognitive screen in 2013: Sensitivity, specificity, LR and PPV**

	**Nigerian outpatients**	**Tanzanian outpatients**	**Tanzanian inpatients**
**AUROC curve**	0.990	0.919	0.917
**Cut-off of ≤ 7**	100% sensitivity	61.5% sensitivity	90.9% sensitivity
	96.3% specificity	93.5% specificity	87.5% specificity
	27.0 LR	9.5 LR	7.3 LR
	75.0% PPV	-	78.9% PPV
**Cut-off of ≤ 8**	100% sensitivity	84.6% sensitivity	93.9% sensitivity
	91.7% specificity	89.1% specificity	81.3% specificity
	12.0 LR	7.8 LR	5.0 LR
	57.1% PPV	-	72.1% PPV

AUROC = area under the receiver operating characteristic.

PPV = positive predictive value.

LR = likelihood ratio.

### The influence of age, gender and education on IDEA cognitive screen performance

Univariate logistic regression models investigating the influence of age, gender, education and the presence of major cognitive impairment on screening performance (outcome variable) are summarised in Table 3. Major cognitive impairment was associated with a cognitive screening score ≤ 7 in all three cohorts, with age as an additional correlate in Tanzanian inpatients. In multivariable analysis, with data for all three cohorts combined (n = 277), female gender, greater age and the presence of major cognitive impairment were independent predictors of low screening score, but education was not, see Table 4. Even after removing gender and age from the model, education level remained a non-significant predictor.

**Table 3:** **Validation of the Identification and Intervention for Dementia in Elderly Africans (IDEA) cognitive screen in 2013: Univariate logistic regression models with dichotomised cognitive screen as the dependent (outcome) variable**

	**Odds ratio (95% CI)**
**Nigerian outpatients**	
Major cognitive impairment present*	-
**Female**	2.82 (0.92 to 8.69)
**No formal education**	3.11 (0.67 to 14.58)
**Age**	
65-69 years	1
70-74 years	1.03 (0.27 to 3.98)
75-79 years	1.88 (0.47 to 7.51)
80-84 years	0.68 (0.07 to 6.26)
85 years and over	1.88 (0.18 to 19.68)
**Tanzanian outpatients**	
**Major cognitive impairment present**	22.93 (4.55 to 115.67)
**Female**	2.25 (0.58 to 8.72)
**No formal education**	2.80 (0.72 to 10.97)
**Age**	
65-69 years	1
70-74 years	6.75 (0.61 to 75.27)
75-79 years	4.00 (0.32 to 50.23)
80-84 years	6.00 (0.46 to 78.56)
85 years and over	7.71 (0.68 to 87.25)
**Tanzanian inpatients**	
**Major cognitive impairment present**	70.00 (17.28 to 283.59)
**Female**	1.00 (0.44 to 2.27)
**No formal education**	1.99 (0.86 to 4.64)
**Age**	
65-69 years	1
70-74 years	4.57 (0.83 to 25.21)
75-79 years	4.31 (0.76 to 24.38)
80-84 years	7.00 (1.17 to 41.76)
85 years and over	14.00 (2.54 to 77.21)

* An odds ratio cannot be calculated due to zero values. Only four subjects, from the 121 in the cohort, were misclassified, all identified as positive on screen, but negative on clinical assessment.

**Table 4:** **Validation of the Identification and Intervention for Dementia in Elderly Africans (IDEA) cognitive screen in 2013: Multivariable logistic regression model with dichotomised cognitive screen as the dependent (outcome) variable**

	**Odds ratio (95% CI)**	**Significance (p)**
**Major cognitive impairment present**	108.82 (36.31 to 326.14)	<0.001
**Female**	3.32 (1.20 to 9.19)	0.021
**No formal education**	1.07 (0.40 to 2.88)	0.895
**Age**		
65-69 years	1	
70-74 years	3.26 (0.83 to 12.74)	0.090
75-79 years	3.52 (0.87 to 14.31)	0.079
80-84 years	5.39 (1.05 to 27.68)	0.044
85 years and over	6.80 (1.58 to 29.21)	0.010

Hosmer and Lemeshow goodness of fit test, χ^2^ (7) = 5.60, p = 0.587.

Nagelkerke R^2^ = 0.66.

### Inter-rater reliability

For inter-rater reliability assessment, 30 patients were seen by raters A and B and 19 by rater C. The median time from the first to the second assessment was 3 days (IQR 2 to 4 days) and the median time from the second to the third assessment was 5 days (IQR 4 to 8 days). The level of agreement between the raters was good. Comparing raters A and B, the ICC was 0.791. For the 19 patients seen by rater C, the ICC was 0.787 compared to rater A and 0.742 compared to rater B. The differences in scores between raters were generally small. Comparing the first two raters, 20 assessments (66.7%) were within one point of each other and 27 (90.0%) within two points of each other. Using ≤ 7 as a cut-off, raters A and B agreed, and would have made the same clinical decision for 28 (93.3%) cases. Likewise, raters A and B agreed on 18 (94.7%) cases, with 13 (68.4%) scores within one point of each other and 18 (94.7%) within two points. Finally, raters A and C agreed on 17 (89.5%) cases, with 15 (78.9%) within one point of each other and 17 (89.5%) within two points.

## Discussion

The IDEA cognitive screen performed well in all three settings, with good internal consistency and inter-rater reliability. The AUROC curve was generally higher than seen during internal validation and fieldwork testing in Tanzania [6]. The screen appeared to be acceptable and culturally appropriate and no one refused assessment.

The sensitivity in Tanzanian outpatients was relatively low, although lower sensitivity in outpatients was expected in this setting. In rural Tanzania, people who have dementia, and who are able to attend outpatient clinics, are likely to be in the early stages of disease. They may therefore be expected to perform relatively well on brief cognitive screening, with the presence of dementia only becoming apparent on more detailed assessment. It is not clear why the screen performed better in Nigerian outpatients than in Tanzanian outpatients. The fact that UCH in Ibadan is a tertiary referral hospital may have played a part, with a broader spread of patients including those with more severe problems, who may be easier to assess cognitively. Further validation work in other settings in Nigeria is merited.

The IDEA cognitive screen performed well in comparison with other major cognitive impairment screening instruments developed for use in populations with low levels of formal education [14,15]. Touré et al. [14] developed the ‘Test of Senegal’ and obtained an AUROC curve of 0.967 on comparison with the DSM-IV-R criteria when blind assessment of 58 cases and 58 controls was carried out. However, the test has 39 questions in total and is therefore too lengthy for use in busy non-specialist hospital settings.

The performance of the IDEA cognitive screen also compares well to tests of cognitive performance validated in HICs [16,17]. The six-item screener comprises three orientation questions and a three-word delayed recall test. It was developed for use in emergency departments and has a sensitivity of 63%, a specificity of 81% and an AUROC curve of 0.77 [18]. The mini-cog comprises a clock-drawing test and a three-word delayed recall test; it has a sensitivity of 75% and specificity of 85% [19]. The general practitioner assessment of cognition (GPCOG) combines the clock-drawing test with items assessing recall and orientation; its sensitivity was 85% and specificity 86% [20]. A review of other brief screening instruments was carried out in 2007 [21].

UK and US good practice guidelines recommend cognitive assessment of older adults in higher-prevalence settings including primary care, and routinely in hospital inpatient and outpatient populations [22]. The existing evidence base strongly suggests that identification of cognitive impairment can improve outcomes and reduce morbidity and mortality through prevention of delirium [23]. Cognitive screening should form a core part of assessment for older hospitalised adults. Surprisingly, even in HICs few of the recommended cognitive screening tools have been validated in general hospital settings [24]. Validation of appropriate cognitive screening methods for hospitalised older adults is therefore needed globally, not only in SSA.

### Limitations

The main limitation of our study is the relatively small number of people in each cohort who had major cognitive impairment. However, the overall number of people with major cognitive impairment (n = 58) was relatively large and results were similar across all settings, allowing data to be combined for multivariable analysis. Any attempt to increase the number of major cognitive impairment cases by assessing only people previously known to have dementia and a group of controls would have reduced the generalisability of our results and may have resulted in substantial bias.

In this hospital-based study, we did not attempt to distinguish patients with delirium from those with dementia. It is not expected that a short screen will be able to distinguish such conditions. The value of carrying out screening is to alert the clinician to cognitive impairment meaning that delirium can be promptly recognised and treated, and possible dementia considered in hospital discharge planning. Without an informant history and follow-up it is difficult to be certain that dementia is present in hospital patients. Despite multiple attempts, it was not always possible to obtain a history from a close relative. Those patients who had a carer tended to be younger and more independent, and were probably less likely to have cognitive impairment. Occasionally carers were distant relatives who were less able to give a detailed history. In the Tanzanian sample, patients who were seriously unwell, and were felt to need admission to the tertiary referral hospital, were transferred, and this is again likely to have led to an underestimate of cases of delirium. In the outpatient settings, resource limitations meant that it was not possible to see all screened patients. However, almost half of those who screened negatively were randomly selected for clinical assessment and any bias is likely to be small. Finally, few people had a birth certificate or had had their birth registered and so a validated method of age estimation was used. The method has been shown to have excellent concordance during validation work in other populations in SSA and we feel that, at a cohort level, any bias will be small.

## Conclusions

The IDEA cognitive screen was administered by non-specialist healthcare workers and performed well in hospital settings in Nigeria and Tanzania. Further testing in other regions of SSA, and in primary care, is an important next step.

## References

[1] Prince M, Guerchet M, Prina M (2013). Policy brief for heads of government: the global impact of dementia 2013–2050.

[2] Dotchin CL, Akinyemi RO, Gray WK, Walker RW (2013). Geriatric medicine: services and training in Africa. Age Ageing.

[3] Bower JH, Zenebe G (2005). Neurologic services in the nations of Africa. Neurology.

[4] Saxena S, Thornicroft G, Knapp M, Whiteford H (2007). Resources for mental health: scarcity, inequity, and inefficiency. Lancet.

[5] Eaton J, McCay L, Semrau M, Chatterjee S, Baingana F, Araya R, Ntulo C, Thornicroft G, Saxena S (2011). Scale up of services for mental health in low-income and middle-income countries. Lancet.

[6] Gray WK, Paddick SM, Kisoli A, Dotchin CL, Longdon AR, Chaote P, Samuel M, Jusabani AM, Walker RW (2014). Development and Validation of the Identification and Intervention for Dementia in Elderly Africans (IDEA) Study Dementia Screening Instrument. J Geriatr Psychiatry Neurol.

[7] Paraiso MN, Houinato D, Guerchet M, Agueh V, Nubukpo P, Preux PM, Marin B (2010). Validation of the use of historical events to estimate the age of subjects aged 65 years and over in Cotonou (Benin). Neuroepidemiology.

[8] Hall KS, Hendrie HC, Brittain HM, Norton JA, Rodgers DD, Prince CS, Pillay N, Blue AW, Kaufert JN, Nath A (1993). The development of a dementia screening interview in 2 distinct languages. Int J Methods Psychiatr Res.

[9] Welsh KA, Butters N, Mohs RC, Beekly D, Edland S, Fillenbaum G, Heyman A (1994). The Consortium to Establish a Registry for Alzheimer’s Disease (CERAD). Part V. A normative study of the neuropsychological battery. Neurology.

[10] Baiyewu O, Unverzagt FW, Lane KA, Gureje O, Ogunniyi A, Musick B, Gao S, Hall KS, Hendrie HC (2005). The stick design test: a new measure of visuoconstructional ability. J Int Neuropsychol Soc.

[11] American Psychiatric Association (1994). Diagnostic and statistical manual of mental disorders.

[12] Paddick S-M, Longdon A, Gray WK, Dotchin C, Kisoli A, Chaote P, Walker R (2014). The association between educational level and dementia in rural Tanzania. Dementia & Neuropsychologia.

[13] Paddick SM, Longdon AR, Kisoli A, Dotchin C, Gray WK, Dewhurst F, Chaote P, Kalaria R, Jusabani AM, Walker R (2013). Dementia prevalence estimates in sub-Saharan Africa: comparison of two diagnostic criteria. Glob Health Action.

[14] Touré K, Coumé M, Ndiaye NND, Thiam MH, Zunzunegui MV, Bacher Y, Gueye L, Tal/Dia A, Ndiaye MM (2008). The test of senegal: a valid and reliable screening tool to assess for dementia in a senegalese elderly population. Afr J Neurol Sci.

[15] Prince M, Acosta D, Ferri CP, Guerra M, Huang Y, Jacob KS, Llibre Rodriguez JJ, Salas A, Sosa AL, Williams JD, Hall KS (2011). A brief dementia screener suitable for use by non-specialists in resource poor settings–the cross-cultural derivation and validation of the brief Community Screening Instrument for Dementia. Int J Geriatr Psychiatry.

[16] Mitchell AJ (2009). A meta-analysis of the accuracy of the mini-mental state examination in the detection of dementia and mild cognitive impairment. J Psychiatr Res.

[17] Larner AJ, Mitchell AJ (2014). A meta-analysis of the accuracy of the Addenbrooke’s Cognitive Examination (ACE) and the Addenbrooke’s Cognitive Examination-Revised (ACE-R) in the detection of dementia. Int Psychogeriatr.

[18] Wilber ST, Carpenter CR, Hustey FM (2008). The Six-Item Screener to detect cognitive impairment in older emergency department patients. Acad Emerg Med.

[19] Wilber ST, Lofgren SD, Mager TG, Blanda M, Gerson LW (2005). An evaluation of two screening tools for cognitive impairment in older emergency department patients. Acad Emerg Med.

[20] Brodaty H, Pond D, Kemp NM, Luscombe G, Harding L, Berman K, Huppert FA (2002). The GPCOG: a new screening test for dementia designed for general practice. J Am Geriatr Soc.

[21] Woodford HJ, George J (2007). Cognitive assessment in the elderly: a review of clinical methods. QJM.

[22] National Institute for Health and Care Excellence (NICE) (2010). Delirium: Diagnosis, prevention and management.

[23] Fong TG, Tulebaev SR, Inouye SK (2009). Delirium in elderly adults: diagnosis, prevention and treatment. Nat Rev Neurol.

[24] Jackson TA, Naqvi SH, Sheehan B (2013). Screening for dementia in general hospital inpatients: a systematic review and meta-analysis of available instruments. Age Ageing.

